# Supplementary material for: Sleep in neurointensive care patients, and patients after brain tumor surgery
Source: PLoS One. 2023 Jun 23;18(6):e0286389. doi: 10.1371/journal.pone.0286389 (PMC10289440; doi:10.1371/journal.pone.0286389)
Supplement: Attachment — Submitted filename: Response.docx [file pone.0286389.s001.docx]

1. Please ensure that your manuscript meets PLOS ONE's style requirements, including those for file naming. The PLOS ONE style templates can be found at 
https://journals.plos.org/plosone/s/file?id=wjVg/PLOSOne_formatting_sample_main_body.pdf and 
https://journals.plos.org/plosone/s/file?id=ba62/PLOSOne_formatting_sample_title_authors_affiliations.pdf

**Response:** We have now adapted to the style requirements

2. Please provide additional details regarding participant consent. In the ethics statement in the Methods and online submission information, please ensure that you have specified what type you obtained (for instance, written or verbal, and if verbal, how it was documented and witnessed). If your study included minors, state whether you obtained consent from parents or guardians. If the need for consent was waived by the ethics committee, please include this information.

**Response:** Details regarding participant consent is inserted in the ethics statement in the Methods and in the online submission information

3. Thank you for stating the following financial disclosure: 
   "EEG equipment was funded by The Swedish Medical Society and Olle Engkvist’s foundation."

Please state what role the funders took in the study.  If the funders had no role, please state: "The funders had no role in study design, data collection and analysis, decision to publish, or preparation of the manuscript." 
If this statement is not correct you must amend it as needed. 
Please include this amended Role of Funder statement in your cover letter; we will change the online submission form on your behalf.

**Response:** This statement is inserted in the cover letter: “The funders had no role in study design, data collection and analysis, decision to publish, or preparation of the manuscript.”

4. Thank you for stating the following in the Acknowledgments Section of your manuscript: 
   "We thank the staff who assisted us in the data collection, and the technicians and engineers at the unit of clinical neurophysiology. EEG equipment was funded by The Swedish Medical Society and Olle Engkvist’s foundation"

We note that you have provided funding information that is not currently declared in your Funding Statement. However, funding information should not appear in the Acknowledgments section or other areas of your manuscript. We will only publish funding information present in the Funding Statement section of the online submission form. 
Please remove any funding-related text from the manuscript and let us know how you would like to update your Funding Statement. Currently, your Funding Statement reads as follows: 
   "EEG equipment was funded by The Swedish Medical Society and Olle Engkvist’s foundation."

Please include your amended statements within your cover letter; we will change the online submission form on your behalf.

**Response:** Thank you. The funding information is removed from the manuscript and placed in the cover letter.

5. In your Data Availability statement, you have not specified where the minimal data set underlying the results described in your manuscript can be found. PLOS defines a study's minimal data set as the underlying data used to reach the conclusions drawn in the manuscript and any additional data required to replicate the reported study findings in their entirety. All PLOS journals require that the minimal data set be made fully available. For more information about our data policy, please see http://journals.plos.org/plosone/s/data-availability.

"Upon re-submitting your revised manuscript, please upload your study’s minimal underlying data set as either Supporting Information files or to a stable, public repository and include the relevant URLs, DOIs, or accession numbers within your revised cover letter. For a list of acceptable repositories, please see http://journals.plos.org/plosone/s/data-availability#loc-recommended-repositories. Any potentially identifying patient information must be fully anonymized.

Important: If there are ethical or legal restrictions to sharing your data publicly, please explain these restrictions in detail. Please see our guidelines for more information on what we consider unacceptable restrictions to publicly sharing data: http://journals.plos.org/plosone/s/data-availability#loc-unacceptable-data-access-restrictions. Note that it is not acceptable for the authors to be the sole named individuals responsible for ensuring data access.

We will update your Data Availability statement to reflect the information you provide in your cover letter.

Additional Editor Comments:
Please carefully read the comments from the reviewers and revise the manuscript in accordance with the cements as Round 2.
[Note: HTML markup is below. Please do not edit.]

**Response:** **Data availability:** The EEG files contain a person-identifying number (person number - a specific individual code identifying each Swedish citizen). Further, the files contain date and time. The person number, and also the information about date and time (together with other data given in the article), may each be enough to identify individual study persons. We have so far not been able to convert EEG data into a format without this identifying information. Therefore, publishing the files is currently not compatible with Swedish legislation and ethical approval (<https://etikprovningsmyndigheten.se/en/>). Data requests may be sent to the head of the Neuro department, Uppsala University Hospital, Uppsala, Sweden.

1. “postoperative patients after brain tumor surgery” contains the meaning of “after surgery” twice. I think “postoperative” is not needed.

**Response:** Thank you for bringing this to our attention. It is now corrected.

2. The following division is a bit strange: “The diagnoses among these patients were subarachnoid haemorrhage(n=16), acute subdural hematoma (n=6), intracerebral haemorrhage (n=4), meningitis (n=4), intraventricular haemorrhage (n=2), cerebellar bleeding (n=1), traumatic subarachnoid haemorrhage (n=1), brain tumour (n=1), stroke (n=1) and cerebellar infarction (n=1)” in the Design and methods. Cerebellar bleeding usually is included intracerebral haemorrhage. Stroke shows cerebral haemorrhage or infarction or subarachnoid haemorrhage. What does stroke (n=1) mean? Please correct it.

**Response:** Cerebellar bleeding is now included intracerebral haemorrhage and STROKE is clarified with thrombectomy.

3. “severely brain-injured” is a keyword in this article. However, it is unclear what “severely brain-injured” means. You should describe the definition of “severely brain-injured” in the Method.

**Response:** Severely brain-injured patients is now defined as patients who were sedated and treated in a ventilator due to their brain damage. This is clarified in the design and methods.

4. It is better to explain the difference between NICU and NIMCU.

**Response:** This is now explained more clearly in the design and methods

5. You mentioned that patients in the NIMCU were prospectively included. If this article is a prospective study or a study using prospectively registered data, you need to describe about obtaining written informed consent in the Ethics.

**Response:** This information is now included in the Ethics.

6. Please show references about the sleep stage (N1, N2).

**Response:** The main reference explaining the scientific ground for the sleep stages definitions is now provided.

7. You mentioned that ”On admission to the NICU the mean Glasgow Coma Scale motor (GCS-M) was 4.8 (range 2-6) and when EEG monitoring was started mean GCS was 4.7 (range 1-6)(16).” Why did you use GCS-motor, instead of GCS? I think using GCS makes it easier to understand the degree of impaired consciousness. You should use GCS or explain the reason to use GCS-motor. And, in “mean GCS was 4.7 (range 1-6),” the minimum of GCS is 3. Is range 1-6 correct? (or do you mean GCS-motor?)

**Response:** We presented GCS-M on the basis that the department where the study was conducted routinely uses the Reaction level scale (RLS). RLS is directly translatable to GCS-M. These patients were all intubated, which made verbal response not possible. Many of these patients are also unable to open their eyes due to swelling. We have data for a complete GCS on arrival but not on EEG start. With all this taken together, we choose to continue reporting GCS-M.

Ref. Starmark JE, Stålhammar D, Holmgren E. The Reaction Level Scale (RLS85). Manual and guidelines. *Acta Neurochir (Wien)*. 1988;91(1-2):12-20. doi:10.1007/BF01400521

8. I think one of the important factors that affect sleep just after surgery is pain. Please consider describing VAS or NRS and analyzing if possible.

**Response:** Unfortunately, we do not have this data available. However, all patients were offered medications, i.e., morphine to control the postoperative pain.

9. Please add more information to the main results. For example, “tumor volume (p=0.61)” can be modified like “tumor volume (XX±XX cm3 in patients with sleep vs YY±YY cm3 in patients without sleep, p=0.61).” Moreover, for emphasis, it would be better to present the main results in a Table.

**Response:** We have now added this information, please see table 3.

10. How about changing “Monitored time in hours” in Table 1 to “EEG Monitored time in hours”? I think this is more understandable.

**Response:** This is corrected.

11. The significance of the following result is not clear because all patients in the NICU group were intubated and controlled at RASS -4: “None of the patients in the NICU had any sleep patterns or sleep phenomena monitored with EEG.” It seems to go without saying that EEG can’t identify the sleep stage under deep sedation or severe disturbance of consciousness. It is required to describe persuasive content in the Discussion while citing past literature.

**Response:** The request to discuss and provide literature references regarding the ability or disability to identify the sleep stages under deep sedation and/or severe disturbance of consciousness is acknowledged and appreciated. New paragraphs pertaining to these issues have been added to the discussion. Some of the text regarding the limitations of EEG to detect sleep in comatose brain injured patients, formerly placed under *Limitations,* have been moved up to the general discussion as this rather belongs to the requested discussion, than constitute a limitation of the study performance. Further, a new paragraph has been added to *Limitations*, as we have identified a source of selection bias that might have contributed to the results (absence of sleep phenomena in NICU patients).

12. You mentioned that “It is important to balance the patients' opportunity to sleep against the risk of not detecting a neurologic deterioration.” I know intuitively that these patients also need sleep. However, it would be better to cite references showing that it is important to get some sleep even in patients requiring neuromonitoring.

**Response:** Thank you for pointing this out, we have now elaborated on this and included relevant references.

13. There are some grammatical mistakes as blow. Please double-check your manuscript.
・The sentence in Aim and objectives of study “Sedation may affect not the ability to sleep, but also the EEG rhythms used to identify sleep” should be modified to “Sedation may affect not ONLY the ability to sleep, but also the EEG rhythms used to identify sleep.”
・In this sentence “All patients who were not responding to commands, Glasgow Coma scale - Motor (GCS-M)(<5, were intubated, sedated and artificially ventilated”, I think “who” is not needed.

**Response:** This is now corrected.

[1] “Introduction”
This study consists of three aims. Therefore, it is better to describe the purpose/aim, methods, and results of each experiment in the manuscript, such as the followings:
[Aim 1]
[Aim 2]
[Aim 3]

**Response:** Thank you for this suggestion. The aim is now divided into three objectives and the rest of the text is corrected to keep that organization as best as possible.

[2] “Abstract”
Please rewrite the abstract so readers can understand this study's essence only by reading the abstract. The authors might want to refer to the following advice;
(1) It is better to describe these three aims in the abstract.
(2) It is better to clarify this study’s outcome.
(3) As for the explanation of two datasets, it is better to state in [Study design] rather than [Background].
(4) It is better to show specific numbers and statistic values in [Results].
(5) Please avoid redundant expressions between [Results] and [Conclusion].

**Response:** Thank you for this advice, we have now implemented most of it.

[3] “Introduction”
Please clarify the clinical relevance or implication of this study in the Introduction.

**Response:** We have now clarified the clinical relevance at the end of the Introduction.

[4] “Methods”
Please describe why the inclusion periods differed for patients in the NICU and NIMCU groups. In addition, please identify the name of the hospital of the NICU group.

**Response:** This information is now added.

[5] “Methods”
Please clarify whether a neurophysiologist was blind to the group of patients reading cEEG monitoring. If not blind, please add that point as a limitation.

**Response:** Information that the EEG reader was not blinded to group has been added to methods and is also problematized under *Discussion*, *Limitations*.

Please separate the tables for the group of the neuro intensive care unit and neuro intermediate care unit. This is because these datasets were used for different aims. In addition, please add the rows of the RASS scale and cause of admission in the table for the neuro intensive care unit group and rows of tumor type, grade, volume, region of brains, past medical history of insomnia, preoperative daily use of medications for insomnia in the table for the group of the neuro intermediate care unit.

**Response:** This information is added except information of past medical history of insomnia, preoperative daily use of medications for insomnia in the table for the group of the neuro intermediate care unit. This is because we unfortunately do not have access to that information

Please add the limitation that the statistical significance of the mean age difference between patients with and without sleep was univariate analysis. This analysis did not consider confounding factors.

**Response:** This limitation is added in the Discussion.

[8] “Methods”
It is helpful for broad readers to briefly explain how the neuro intensive care unit and neuro intermediate care unit are defined or the inclusion criteria for each ward.

**Response:** This is now explained more clearly in the design and methods

[9] “typo”
Abstract: not no fall asleep -> not fall asleep

**Response:** This is corrected

3. Have the authors made all data underlying the findings in their manuscript fully available?

The [PLOS Data policy](http://www.plosone.org/static/policies.action#sharing) requires authors to make all data underlying the findings described in their manuscript fully available without restriction, with rare exception (please refer to the Data Availability Statement in the manuscript PDF file). The data should be provided as part of the manuscript or its supporting information, or deposited to a public repository. For example, in addition to summary statistics, the data points behind means, medians and variance measures should be available. If there are restrictions on publicly sharing data—e.g. participant privacy or use of data from a third party—those must be specified.

Reviewer #1: Yes

Reviewer #2: Yes
